# Public Knowledge and Attitudes towards Bystander Cardiopulmonary Resuscitation in China

**DOI:** 10.1155/2017/3250485

**Published:** 2017-03-07

**Authors:** Meng Chen, Yue Wang, Xuan Li, Lina Hou, Yufeng Wang, Jie Liu, Fei Han

**Affiliations:** Department of Anesthesiology, The Third Affiliated Hospital, Harbin Medical University, Harbin, Heilongjiang 150081, China

## Abstract

The rate of bystander CPR is much lower in China than in developed countries. This survey was implemented to assess the current status of layperson CPR training, to analyze the willingness of bystanders to perform CPR, and to identify barriers to improving bystander CPR rates. The questionnaire included individual information, current status of bystander CPR training, and individual's willingness and attitude towards performing CPR. There were 25.6% laypersons who took CPR training. The majority (98.6%) of laypersons would perform CPR on their family members, but fewer laypersons (76.3%) were willing to perform CPR on strangers. Most respondents (53.2%) were worried about legal issues. If laws were implemented to protect bystanders who give aid, the number of laypersons who were not willing to perform CPR on strangers dropped from 23.7% to 2.4%. An increasing number of people in China know CPR compared with the situation in the past. CPR training in China is much less common than in many developed countries. The barriers are that laypersons are not well-trained and they fear being prosecuted for unsuccessful CPR. More accredited CPR training courses are needed in China. The laws should be passed to protect bystanders who provide assistance.

## 1. Introduction

Cardiac arrest is a substantial public health problem. Data from previous studies suggest that more than 3 million sudden cardiac deaths occur worldwide every year [1, 2], and survival is lower than 8% [3]. Unfortunately, 544,000 sudden cardiac deaths occur in China each year with survival of less than 1% [4]. Survival from sudden cardiac arrest in China is much lower than in many countries. Immediate bystander cardiopulmonary resuscitation (CPR) increases out-of-hospital cardiac arrest (OHCA) survival by twofold to threefold [5–7]. The chance of surviving OHCA falls by 7%–10% per minute without intervention [8].

The proportion and intensity of bystander CPR training vary in different countries. The main reason of the difference on bystander CPR training between countries is because of the various education and training system, such as CPR training as a part of the middle school curriculum and driver's license acquisition [9–11]. The willingness of the laypersons in different countries to learn and perform CPR is also very important. No time and no interest to learn CPR, afraid of doing something wrong, a fear of legal liability, and other reasons are obstacles limiting bystander to learn and perform CPR [12–14]. More than half of the students in the United States learned CPR and automated external defibrillator [15]. In Norway, 89% secondary school students attended CPR training [12]. Seventy percent of people in Japan learned CPR, and 30% of people learned CPR more than two times [10]. However, CPR training among Chinese students is 27%, which was much lower than in developed countries [10, 12, 15, 16].

One or two decades ago, there were few CPR training for the public and students in China. Accredited CPR training courses were only for medical staff or emergency medical service related professions, such as firefighter. With the development of China and the improvement of civilization of Chinese society, CPR training courses by medical organization, television, Internet, newspaper, and other channels are developing recent years for the public and university students, but it is still not systematic. Because of the big population of Chinese people, organized training by government such as including the training as a part of senior school curriculum and driver's license acquisition is needed to be established. Also, it is important to increase the willingness of the people to supply help to cardiac arrest victims. Several surveys were conducted to investigate the knowledge and attitudes of bystander CPR on Chinese students [16, 17]. However, few studies about bystander CPR towards the public were done in China. This survey was implemented to assess current status and effects of bystander CPR training on the public, to explore the willingness of bystanders to perform CPR, and to identify barriers to improve bystander CPR rate in China.

## 2. Methods

This study was approved by the ethics committee of the Third Affiliated Hospital, Harbin Medical University. The data of this survey was acquired by questionnaires distributed to the public of China through the Internet.

### 2.1. Questionnaire Design and Distributing

The questionnaire consisted of three sections with a total of 19 questions including individual information, current status and effects of CPR training, attitude on CPR training, and willingness to providing help in emergency situation. The questionnaires were released at https://www.sojump.com from May 9 to 19, 2014. This website is one of the largest websites which provide a platform for researchers who design and release questionnaires to make all kinds of survey on the public of China. The website has over 2.6 million volunteer members in China. The questionnaires were distributed randomly to volunteer members by email invitation. There was no conflict of interest between the volunteer members and the survey. The website automatically screened IP addresses to ensure that the questionnaire was answered only once from each IP address. The website automatically ruled out answers if the feedback for the whole questionnaire was less than 2 minutes or more than 30 minutes. The time limitations are calculated automatically by the website of https://www.sojump.com according to the number and content of the questions of the questionnaire. The website provided the email address and IP address of each returned questionnaire to make sure each answered questionnaire had a reachable respondent and was credible.

### 2.2. Data Analysis

Percentages were calculated for the frequencies. The difference between groups was analyzed with Chi-square tests or Fisher exact tests. *P* values < 0.05 were considered significantly different. All statistics were processed with SPSS for Windows 17.0 (SPSS Inc., Chicago, IL, USA).

## 3. Results

A total of 2102 answered questionnaires were collected by May 19, 2014. Eight questionnaires were ruled out because of obvious contradictive answers. Among the valid respondents (Table 1), 87.9% were laypersons (compared with medical related person). The questionnaires answered by laypersons (1841) were selected for final analysis in this study.

### 3.1. Individual Information

Among the layperson respondents, 49.2% were male and 50.8% were female, 99.6% were between 18 and 60 years old, and 78.5% had a bachelor's degree or above, 17.5% had associate's degree, and 4.0% had educational level of lower than associate's degree.

### 3.2. Current Status and Effects of CPR Training

Among the layperson respondents, 90.1% understood what is CPR and 25.6% were trained by CPR courses (Table 2). However, among the trained laypersons, 50.8% knew the standard CPR procedure and believed they had the ability to perform CPR, and 49.2% knew the procedure only but they did not believe they had the ability to perform CPR on victims (Table 3). The top three reasons for not attending CPR training courses were not knowing where to take the training (54.7%), a lack of time (20.1%), and a lack of concern (10.7%). Sixty-six point one percent of laypersons believed they had the ability to learn and perform CPR, and 79.1% believed that a lifeless person without breath and/or heartbeat could be saved. Female had less confidence than male (*P* < 0.001). The favorable methods for the laypersons to learn CPR were medical staff teaching (44.9%) or the qualified CPR training courses (27.8%). Fifty-six point one percent of laypersons were willing to pay for the CPR training courses.

### 3.3. Willingness to Perform CPR

If there was a cardiac arrest victim, 59.5% of laypersons stated that they would ask for help and perform CPR to rescue the victim simultaneously, 22.8% chose to ask for help only and wait for rescue, 14.9% chose to perform CPR only, and 2.6% did not know what to do (Table 4). In case of cardiac arrest, 54.0% of laypersons expected to be resuscitated by anybody nearby immediately and 46.0% of laypersons expected to be resuscitated by the medical staff or trained people only. The majority (98.7%) of laypersons chose to perform CPR on their family members when they were confronted with cardiac arrest. However, fewer laypersons (76.3%) were willing to perform CPR on strangers in comparison with family members. There was significant difference in the attitudes of the layperson on family members compared with strangers (*P* < 0.05). With hesitation to rescue strangers, 53.2% of the respondents were worried about legal issues, and 44.4% worried about inadequate knowledge. Interestingly, the most important thing that man worried about was legal issues (59.3%), and their second concern was inadequate knowledge and skill in performing CPR (38.0%). By contrast, woman worried more about inadequate knowledge and skill in CPR (50.5%), and their second concern was legal issues (47.3%). There was significant difference in the most concerned thing when performing CPR between man and woman (*P* < 0.001). Most people (58.9%) believed they would not prosecute bystanders for liability if bystander CPR on their family members failed, 13.7% would, and 27.3% were not sure. Woman had less willingness on prosecuting for liability on bystanders whose CPR failed compared with man (*P* < 0.001). In case laws were implemented to protect bystander who gave aid, the number of laypersons who were not willing to perform CPR on strangers dropped from 23.7% to 2.4% (*P* < 0.001).

## 4. Discussion

Bystander CPR is the most significant factor for OHCA survival. Bystander CPR rates are below 50% on average, and the rates vary between different countries according to reports [18–20]. Bystander CPR occurs less than 6% of OHCA in China [21]. For the purpose of improving the rate of bystander CPR and the ability of the layperson to perform CPR, this study was performed to find out the current status and effects of CPR training, to understand attitudes of layperson towards bystander CPR, and to uncover the barriers to improving bystander CPR rates in China. This study showed 90% of laypersons understood what is CPR in China. One-fourth of the laypersons were trained by different kinds of CPR courses. Thirteen percent of laypersons knew the standard CPR procedure and believe they had ability to perform CPR. The primary reason for layperson not learning CPR was that they did not know where to find a CPR training course. When confronting possible cardiac arrest victims, almost all laypersons chose to perform CPR on their family members, whereas a significantly decreased percentage of respondents chose to perform CPR on strangers. Bystander CPR provided by citizens is fundamentally based on goodwill and conscience towards strangers. The awareness should be emphasized by the appropriate education system. Furthermore, the primary reasons preventing bystanders from supplying help were inadequate knowledge and legal issues. It is important to set up more accredited CPR training courses in China. There are no laws to protect bystanders who are supplying help from being prosecuted in case CPR fails in China. If laws were implemented to protect bystanders who give aid, the number of laypersons who were not willing to perform CPR on strangers dropped 90%.

This study showed that the number of people who understood what is CPR was greatly extended than 7 years ago (90% versus 55%) [16]. However, layperson CPR training (25.6%) in China is still lower than most developed countries, such as Sweden (54.4%) [22], Slovenia (69.4%) [11], United States (54.1%) [23, 24], Australia (58%) [25], and New Zealand (76%) [26]. In Norway, basic life support (BLS) training was up to 89% among secondary school students [12]. In Japan, more than 70% people attended CPR training [10, 27]. Nevertheless, 26.8% of laypersons attended BLS training in Korea and 21% in Hong Kong, which is similar to the data in our study [13, 28].

This study showed that the primary reasons for layperson not attending the CPR training courses were “not knowing where are the CPR training courses” and “a lack of time and concern.” The cost was not considered to be a major problem to learn CPR by the respondents. The reasons for not taking CPR training courses were the same as those noted in Belgium [14]. Although CPR training was very common, the same problems were also reported in Norway [12]. Slovenia has a rather high percentage of CPR-trained layperson, which is related to mandatory CPR training during driver's license acquisition [11]. Approximately 50 years ago, BLS was recommended as part of the school curriculum with compulsory resuscitation training in Norwegian schools [12]. CPR training is part of school law for high school graduation in approximately 20 states in the USA at present. In Japan, the percentage of high school students who had previous CPR training courses was 59% [10]. Only 27% of Chinese middle school students learned CPR from television and books, but not from accredited or formal CPR training courses [16]. University students in nonmedical related specialties in China were not required to take CPR theory or technique practice training [17]. These reports indicated that compulsory CPR training organized by government was recommended to be added to the educational system of middle school and college curriculum and driver's license acquisition to improve the ability of bystanders to perform CPR in China, which had been established many years ago in Norway, United State, Japan, and Slovenia who are good performers of bystander CPR.

Nearly half of the laypersons who were trained of CPR believed they knew the procedure only but could not perform CPR which suggested that the quality of CPR training was not satisfactory. Berden et al. reported that reinstruction at six-month intervals is needed to maintain adequate skills in CPR [29]. Similar situations were also reported in other surveys [10, 12, 13]. All Norwegian children went through one BLS course during middle school, but only 73% of the students could recall what they learned about BLS training after several years [12]. In Japan, approximately 30% of respondents attended CPR training more than twice, but some of them believed they had poor knowledge and lacked the ability to perform CPR [10]. CPR training was shown to play an important role in improving recognition of a patient with cardiac arrest [11]. Repeated and effective CPR training increased bystander's confidence and willingness to perform CPR [10, 13]. Multiple CPR training is needed for the public to provide high-quality CPR.

In this study, nearly all laypersons chose to perform CPR on their family member. However, significantly fewer respondents were willing to perform CPR on strangers. In this hesitancy to resuscitate strangers, legal issue was the number one concern of respondents. The second bystander's scruple was inadequate knowledge and skill in performing CPR. These findings were consistent with a survey from Taiwan in 2013 [30]. The first two concerns among the Norwegian students were little knowledge of BLS and a fear of harming the victims [12]. Poor knowledge/performance was the most important anxiety among Japanese teachers/students [10]. The most important thing that Chinese respondents worried about was legal issues, but poor knowledge was prior concern among laypersons of Norwegian. One of the major reasons of this difference was because there were laws to protect people who provide aid to victims in many countries, such as United States, Canada, and many European countries. These laws protect people who supplied help, as in the case of the “Good Samaritan” Law, which offers legal protection to people who give reasonable assistance to those who are injured or ill, encourage people to offer assistance, and reduce bystander hesitation to assist for fear of being sued or prosecuted for unintentional injury or wrongful death [31]. However, no relevant laws have been passed to protect people who provide help from prosecution because of failed resuscitation in China. A similar report was also published in Korea in 2010, showing that, among the respondents who declined to perform standard CPR, the majority of them cited a fear of legal liability before “Good Samaritan” legislation was passed by the government of Korea [13]. According to our study, if laws were implemented to protect bystanders who gave aid, more respondents were willing to provide help to strangers. Interestingly, males worried more about legal issues, while females concerned more about inadequate knowledge and skill of CPR. Furthermore, males reported significantly higher confidence than females in learning and performing CPR in our study, which was consistent with a previous report [12]. On the basis of these reports and the results of this study, the laws should be passed in China to encourage the people and protect the person who supply help to victims without any delay or hesitation.

## 5. Limitations 

Our study has several limitations. First, this survey was released through a website to respondents who were registered volunteer members. The person who answered the questionnaire usually paid more attention to CPR. Therefore, there was a volunteer bias in this study. Second, only 2102 questionnaires were collected in this study, so the results of this survey are difficult to be generalized to the greater Chinese population. However, the volunteer people who finished the survey are from all over the country, including all ages with the ability to answer the questionnaires, and including all kinds of education levels and occupations. This study still showed very important information about the current status of bystander CPR training on Chinese average people in all ages and occupations, because previous studies on bystander CPR training in China were almost done on Chinese students. Third, most of respondents were between 18 and 60 years old and many of them had bachelor's degree or above. The education of the people who answered the questionnaire is above the average level of Chinese. These individuals might have more motivation and opportunity to learn and practice CPR. Thus, the data presented in this study might be optimal than the reality.

## 6. Conclusions

An increasing number of people understood what is CPR compared with the situation in the past with the rapid developing of the society of China. However, layperson CPR training and bystander CPR rate in China are still less common than in many developed countries. People are willing to learn and perform CPR to victims. The barriers are that laypersons are not well-trained and have fear of being prosecuted responsibility for unsuccessful CPR. Because repeated and effective CPR training are important to increase bystander's confidence and willingness to perform CPR, multiple CPR training is needed for the public to provide high-quality CPR. Besides, the awareness should be emphasized by the appropriate education system to motivate people to supply help in emergency situation. The laws protecting laypersons who provide reasonable aid are needed to be made to encourage people to offer assistance without any hesitation in China.

## Figures and Tables

**Table 1:** Characteristics of the respondents.

	All	Medical related person	Layperson
Respondents	2094 (100.0%)	253 (12.1%)	1841 (87.9%)
Gender			
Male	1005 (48.0%)	100 (39.5%)	905 (49.2%)
Female	1089 (52.0%)	153 (60.5%)	936 (50.8%)
Age, y			
<18	3 (0.1%)	0 (0%)	3 (0.2%)
18–25	494 (23.6%)	51 (20.2%)	443 (24.1%)
26–45	1500 (71.6%)	196 (77.5%)	1304 (70.8%)
46–60	93 (4.4%)	6 (2.4%)	87 (4.7%)
>60	4 (0.2%)	0 (0%)	4 (0.2%)
Education level			
<associate's degree	76 (3.6%)	3 (1.2%)	73 (4.0%)
Associate's degree	359 (17.1%)	37 (14.6%)	322 (17.5%)
Bachelor's degree or above	1659 (79.2%)	213 (84.2%)	1446 (78.5%)

**Table 2:** Current status and attitude of layperson towards CPR training.

Layperson responses	All	Male	Female	*P* value
Understanding what is CPR				0.645
Yes	1658 (90.1%)	818 (90.4%)	840 (89.7%)	
No	183 (9.9%)	87 (9.6%)	96 (10.3%)	
CPR training				0.110
Yes	472 (25.6%)	247 (27.3%)	225 (24.0%)	
No	1369 (74.4%)	658 (72.7%)	711 (76.0%)	
Reasons for not attending CPR training				0.044
Do not know where the training is	749 (54.7%)	352 (53.5%)	397 (55.8%)	
Lack of time	275 (20.1%)	148 (22.5%)	127 (17.9%)	
Not concerned	147 (10.7%)	74 (11.2%)	73 (10.3%)	
Costs	118 (8.6%)	56 (8.5%)	62 (8.7%)	
Others	80 (5.8%)	28 (4.3%)	52 (7.3%)	
The way to learn CPR				<0.001
Teaching by medical staff	826 (44.9%)	414 (45.7%)	412 (44.0%)	
Accredited CPR training courses	511 (27.7%)	210 (23.2%)	301 (32.2%)	
TV or internet	290 (15.8%)	164 (18.1%)	126 (13.5%)	
Health education lectures	161 (8.7%)	91 (10.1%)	70 (7.5%)	
Books, newspapers, and magazines	35 (1.9%)	16 (1.8%)	19 (2.0%)	
Others	18 (1%)	10 (1.1%)	8 (0.9%)	
Do you want to pay for the qualified and professional CPR training				0.007
Yes	1032 (56.1%)	534 (59.0%)	498 (53.2%)	
No	258 (14.0%)	131 (14.5%)	127 (13.6%)	
Uncertain	551 (29.9%)	240 (26.5%)	311 (33.2%)	
Do you believe you have ability to learn and perform CPR				<0.001
Yes	1217 (66.1%)	627 (69.3%)	590 (63.0%)	
No	151 (8.20%)	83 (9.2%)	68 (7.3%)	
Uncertain	473 (25.7%)	195 (21.5%)	278 (29.7%)	
Do you believe a lifeless person without breath and/or heartbeat can be saved				<0.001
Yes	1456 (79.1%)	738 (81.5%)	718 (76.7%)	
No	61 (3.3%)	38 (4.2%)	23 (2.5%)	
Uncertain	324 (17.6%)	129 (14.3%)	195 (20.8%)	
Top 5 professions to learn CPR				0.278
Medical staff	1786 (97.0%)	873 (96.5%)	913 (97.6%)	
Firefighter	1578 (85.7%)	785 (86.7%)	793 (84.7%)	
Police officer	1396 (75.8%)	708 (78.2%)	688 (73.5%)	
Driver and steward	1217 (66.1%)	578 (63.9%)	639 (68.3%)	
Tour guide	913 (49.6%)	426 (47.1%)	487 (52.0%)	

*P* < 0.05, statistically significant difference between genders for each question.

**Table 3:** The effects of layperson CPR training.

Layperson responses	All	Male	Female	*P* value
Trained				0.469
Know procedure and can perform CPR	239 (13.0%)	129 (14.3%)	110 (11.8%)	
Know the procedure but cannot perform CPR	233 (12.6%)	118 (13.0%)	115 (12.3%)	
Not trained				0.939
Know the procedure	322 (17.5%)	152 (16.8%)	170 (18.2%)	
Know a little	733 (39.8%)	354 (39.1%)	379 (40.5%)	
Unknown	314 (17.1%)	152 (16.8%)	162 (17.3%)	

*P* value, compared between genders for each question.

**Table 4:** Layperson responses to the hypothetical cardiac arrest scenarios.

Layperson responses	All	Male	Female	*P* value
If you find someone who has cardiac arrest, you will				0.002
Ask for help and perform CPR	1096 (59.5%)	562 (62.1%)	534 (57.1%)	
Ask for help only	420 (22.8%)	174 (19.2%)	246 (26.3%)	
Perform CPR only	274 (14.9%)	149 (16.5)	125 (13.4)	
Do not know how to do	48 (2.6%)	19 (2.1%)	29 (3.1%)	
Others	3 (0.2%)	1 (0.1%)	2 (0.2%)	
If you experience cardiac arrest in public area, the person you wish to perform CPR on you				0.806
Bystander	994 (54.0%)	486 (53.7%)	508 (54.3%)	
Medical staff or trained people only	847 (46.0%)	419 (46.3%)	428 (45.7%)	
When you witness a family member/stranger confronting cardiac arrest, you will perform CPR to				0.094
Both of them	1395 (75.8%)	696 (76.9%)	699 (74.7%)	
Family member only	421 (22.9%)	192 (21.2%)	229 (24.5%)	
Stranger only	10 (0.5%)	6 (0.7%)	4 (0.4%)	
Neither	15 (0.8%)	11 (1.2%)	4 (0.4%)	
While performing CPR on a stranger, you will worry about				<0.001
Legal issues	980 (53.2%)	537 (59.3%)	443 (47.3%)	
Inadequate knowledge and skill of CPR	817 (44.4%)	344 (38.0%)	473 (50.5%)	
Disease transmission	33 (1.8%)	18 (2.0%)	15 (1.6%)	
Others	11 (0.6%)	6 (0.7%)	5 (0.5%)	
If a family member has cardiac arrest, but bystander CPR failed, will you prosecute for liability				<0.001
Yes	252 (13.7%)	140 (15.5%)	113 (12.1%)	
No	1085 (58.9%)	560 (61.9%)	525 (56.1%)	
Uncertain	503 (27.3%)	205 (22.7%)	298 (31.8%)	
If laws were implemented to prevent prosecuting for liability, will you perform CPR on strangers				0.029
Yes	1629 (88.5%)	812 (89.7%)	817 (87.3%)	
No	45 (2.4%)	26 (2.9%)	19 (2.0%)	
Uncertain	167 (9.1%)	67 (7.4%)	100 (10.7%)	

*P* < 0.05, statistically significant difference between genders for each question.
